# Acutherapy for Knee Osteoarthritis Relief in the Elderly: A Systematic Review and Meta-Analysis

**DOI:** 10.1155/2019/1868107

**Published:** 2019-02-17

**Authors:** Zidan Gong, Rong Liu, Winnie Yu, Thomas Kwok-Shing Wong, Yuanqi Guo, Yue Sun

**Affiliations:** ^1^Institute of Textiles and Clothing, The Hong Kong Polytechnic University, Hong Kong; ^2^GINGER Knowledge Transfer and Consultancy Limited, Hong Kong; ^3^Pok Oi Hospital-The Chinese University of Hong Kong Chinese Medicine Centre for Training and Research (Shatin), Hong Kong

## Abstract

**Purpose:**

This systematic review and meta-analysis was conducted to investigate the effects of various acutherapies on knee osteoarthritis (KOA) relief in the elderly.

**Methods:**

Five databases were accessed from inception to July 2017 for searching randomized controlled trials (RCTs) on acutherapy for KOA relief in the elderly. Data were pooled after trial quality assessment for meta-analysis. Outcomes were the scores of knee pain, knee stiffness, and physical function accessed by Western Ontario and McMaster Universities Osteoarthritis (WOMAC) Index.

**Results:**

17 RCTs including 4774 subjects were included. The results indicated that acutherapy significantly affected knee pain (standardized mean difference, i.e., SMD = - 0.73, [95% CI, -0.98 to -0.47], P <0.001), knee stiffness (SMD = -0.66, [95%CI, -0.85 to -0.47], P <0.001), and physical function (SMD = -1.56, [95%CI, -2.17 to -0.95], P<0.001) when compared with control condition without intervention of any acutherapy. Moreover, acutherapy was more effective than corresponding sham (placebo) intervention applied on nonacupoints (SMD = -0.16, [95% CI, -0.32 to -0.01], P = 0.04). However, no significant differences were found on treatment effects between acutherapy and sham acutherapy at the same acupoints (SMD= - 0.09, [95%CI, -0.40 to 0.21], P = 0.55).

**Conclusions:**

Acutherapy was an effective approach for KOA relief in the elderly. The selection of acupoints position could be a crucial factor that influences the treatment efficacy of acutherapy.

## 1. Introduction

Knee osteoarthritis (KOA) is a frequent chronic crippling joint disease especially in the elderly which causes considerable pain, stiffness, and lower extremities disability that significantly affect self-independence and quality of life of the patients [1–5]. For each individual, the lifetime risk of symptomatic KOA was 44.70%, even higher among those who were obese or with a history of knee injury [6, 7]. To relieve symptomatic KOA of the elderly, nowadays acutherapy has been widely recognized and accepted as an alternative therapeutic approach in clinical practice. Acutherapy belongs to the Traditional Chinese Medicine (TCM) based on the principle of acupoint stimulation across meridians through a wide range of modalities such as needle acupuncture, laser acupuncture, acupressure, electroacupuncture, moxibustion, etc. This approach has been used for centuries and demonstrated to be safe, convenient, and effective in treatment of musculoskeletal and connective tissue disorders [8, 9]. However, studies on its treatment of KOA in the elderly are still sparse and inconclusive. The present study aims to perform a systematic review and meta-analysis to evaluate effectiveness of various acutherapies on relief of symptomatic KOA in the elderly. The comparative study on the effects of acutherapy was conducted by comparing with control condition without intervention of any acutherapy, and with different types of sham acutherapies (placebo interventions) focusing on aspects of knee pain, knee stiffness, and physical function.

## 2. Method

### 2.1. Search Strategy

A systematic search was performed for academic literature following the Preferred Reporting Items for Systematic Review and Meta-Analysis (PRISMA) statement [27]. Five databases including Medline, Cochrane Library, Scopus, CINAHL, and Chinese Academic Journals were accessed for searching from inception to July 2017 using the keywords of “acu-therapy/acupuncture/acupressure/acupoint/acu-treatment”, “knee osteoarthritis/knee OA/KOA/osteoarthritis on knee” and “elderly/ older adults/ old people/ aging group/ senior citizen”. In addition, hand search was conducted among the references of the included studies to identify other researches that were missed by electronic search.

### 2.2. Eligibility Criteria

To select studies for systematic review and meta-analysis, inclusion criteria were adopted as follows: (1) randomized controlled trial (RCT); (2) acutherapy as an intervention; (3) subjects who have been diagnosed with symptomatic KOA; (4) subjects who are adults with the average age of at least 50 years. Selected potential trials then were excluded according to the following exclusion criteria: (1) duplications; (2) sample size less than 30; (3) subjects who have other serious diseases such as cancer, stroke, Alzheimer, etc.; (4) acutherapy which acts as an adjuvant therapy of other treatments for KOA management in trials; (5) insufficient data for meta-analysis; and (6) others: trials described too generally or conducted by unconventional or unorthodox methodology.

### 2.3. Quality Assessment

Methodological quality of the identified RCTs was assessed by two authors (Gong Zidan and Sun Yue) according to the modified five-point Jada Scale [28, 29]. Five items were established in the scoring system, which are described as (1) randomized; (2) appropriate randomization method; (3) intervention blinded to the subject; (4) intervention blinded to the evaluator; and (5) description of withdrawals or dropouts. Each item was assessed as either “Yes” or “No”. Each “Yes” would score a single point while each “No” would score a zero point. Score of the modified Jada Scale could range from 0 to 5 for each trial that the higher score indicates lower risk of bias and higher methodological quality. Only those trials with the score of three or above were included for meta-analysis.

### 2.4. Data Extraction and Analysis

For each included RCT, the information including authors, study setting, sample size, subject characteristics, acutherapy modes, treatment protocol, acupoints used, outcome measures, and effectiveness, was carefully reviewed. When various outcome measures were adopted by those trials, the most commonly and frequently used measures were selected to extract data for analysis. The statistical analysis was conducted using the Review Manager 5.3. The extracted data was analyzed in 95% confidence intervals (95% CI) with the statistical significance set at P < 0.05. Multiple scaled data for the same outcome measurement of two parallel arms in each subgroup were compared at the endpoint of intervention. I square (I^2^) test was used to address heterogeneity among the included studies. Random-effects model was applied for data analysis if I^2^ > 50%; otherwise, the fixed-effect model was applied.

## 3. Result

### 3.1. Study Selection and Characteristics

**Figure 1:**
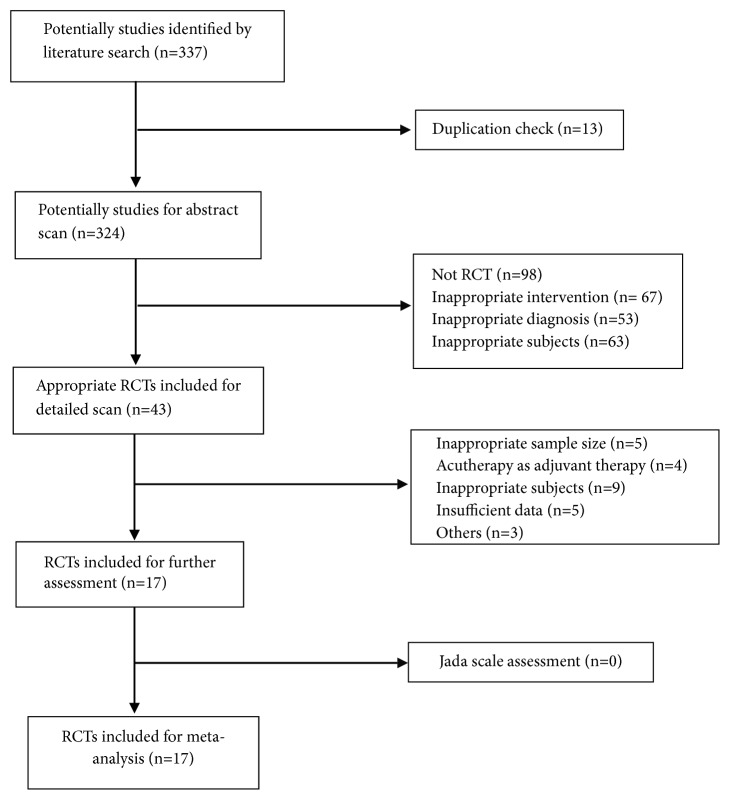
Study search and selection flow.

A total of 17 RCTs including 4774 subjects were identified following the selection flow in Figure 1. The remained 17 RCTs were assessed by the Jada Scale Scoring system with the results presenting in Table 1 to ensure a high methodological quality of the included trails for meta-analysis.  Table 2 summarized the characteristics of the included trials with the sample size ranging from 40 to 1039 and treatment period varying from 2 weeks to 12 months. The most frequently adopted research measures for KOA condition record is the WOMAC, which could be pooled for data extraction in this study. Majority of the trials used acupuncture as the intervention in experimental groups for KOA relief to compare effects with either sham acupuncture groups or with control groups, even with other treatments [11–22]. Other acutherapy modalities included acupressure [9], laser acupuncture [25, 26], electroacupuncture [11, 12, 17], and moxibustion [23, 24].

### 3.2. Meta-Analysis

#### 3.2.1. Acutherapy versus Usual Care

**Figure 2:**
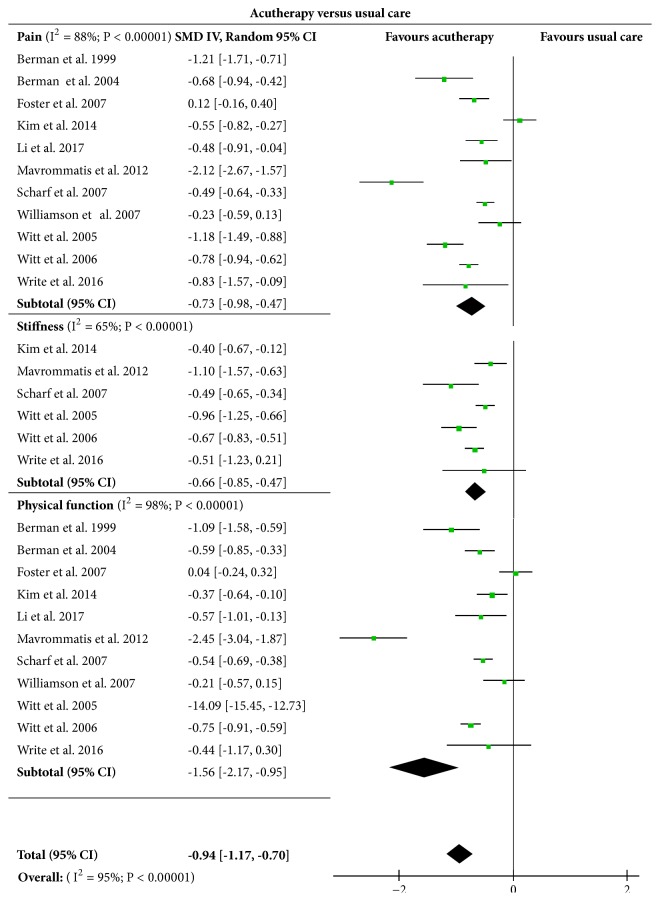
Effects of acutherapy compared with usual care.

The eleven included trials [10–17, 19, 21, 23] involved 2540 subjects who were assessed on the effects of acutherapy on knee pain, knee stiffness, and physical function as compared with that of usual care. Usual care here means that the subjects involved in the control group maintained their previous usual condition, e.g., medication, education, and daily exercise, etc., without intervention of any acutreatment. Results in the Figure 2 indicated a significant difference existing between the experimental acutherapy groups and the control groups in terms of knee pain (SMD = -0.73, [95%CI, -0.98 to -0.47], P <0.001), knee stiffness (SMD = -0.66, [95%CI, -0.85 to -0.47], P <0.001), and physical function (SMD = -1.56, [95%CI, -2.17 to -0.95], P<0.001). The overall test results indicated that acutherapy exerted significant impact on relief of KOA when compared with usual care (SMD= -0.94, [95%CI, -1.17 to -0.70], P<0.001).

#### 3.2.2. Acutherapy versus Sham Acutherapy

**Figure 3:**
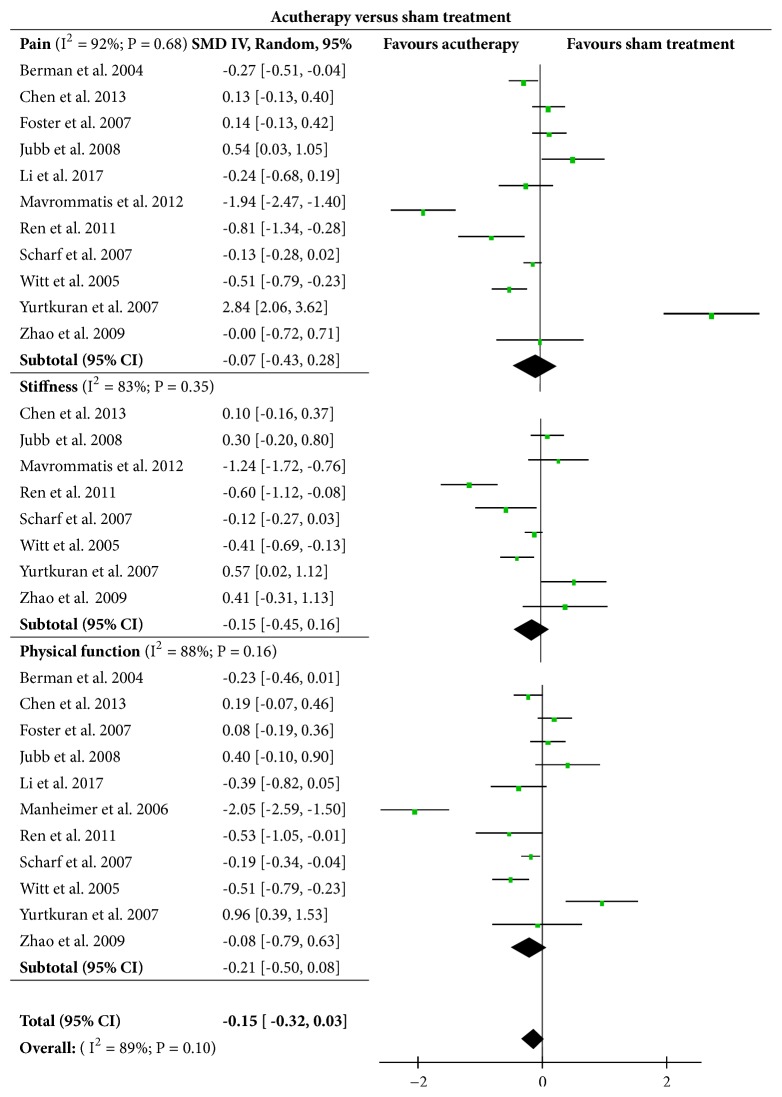
Effects of acutherapy compared with sham acutherapy intervention.

Eleven of the included trials [10, 12, 13, 15, 18–21, 24–26] involving 1982 subjects were analyzed to compare treatment effects between acutherapy and sham acutherapy on knee pain, stiffness, and physical function status. Results in Figure 3 suggested that the acutherapy had no significant clinical effect on improvements of knee pain (SMD = -0.07, [95% CI, -0.43 to 0.28], P = 0.68), knee stiffness (SMD = -0.15, [95% CI, -0.45 to 0.16], P = 0.35), and physical function (SMD = -0.21, [95% CI, -0.50 to 0.08], P = 0.16) when compared with sham intervention. The overall test result showed that no significant difference in effects was found between the acutherapy and sham acutherapy (SMD = -0.15, [95% CI, -0.32 to 0.03], P = 0.10).

#### 3.2.3. Acutherapy versus Sham Acutherapy on the Same Acupoints

**Figure 4:**
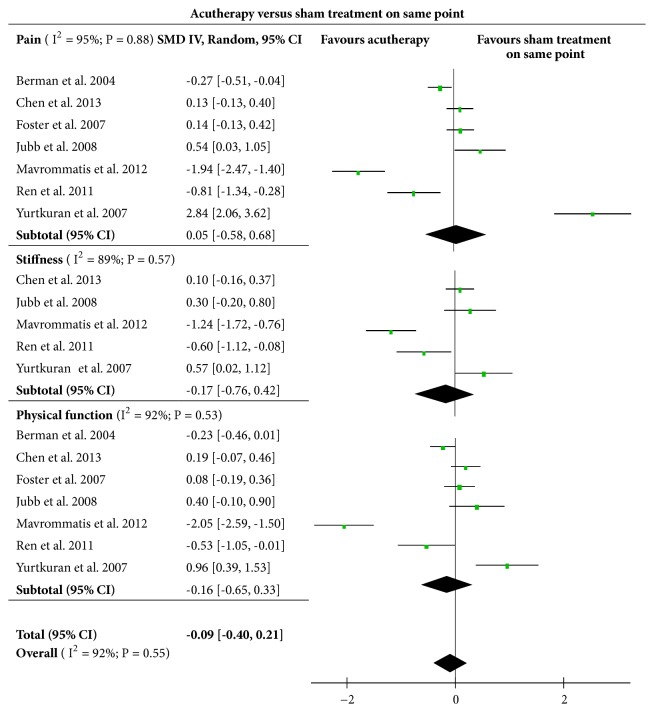
Effects of acutherapy compared with sham intervention on the same acupoints.

Among those trials which made comparison on the effects between acutherapy and sham acutherapy groups, seven studies [12, 15, 18–20, 24, 26] applied sham intervention on the same acupoints as those used in true acutherapy. Five of these trials set up sham groups by conducting noninvasive acupuncture using nonpenetrating needle to achieve slighter stimulations on the skin. Additionally, Ren et al. [24] adopted boned moxibustion cone on EX-LE 5, ST35 and trigger points in experimental group while applying nonmoxibustion cone with similar appearance to the same acupoints as the sham intervention. Aculaser therapy was used in the experimental group in the study conducted by Yurtkuran et al., [26] with 10 mW/cm^2^ power density, 4 mW output power, 0.4 cm^2^ spot size, and 0.48 J dose per session on the acupoint of SP9. To perform a sham intervention, the instrument (infrared 27 GaAs diyode laser instrument, Roland Serie Elettronica Pagani) was switched off and subjects could see the red light of the device for convincing. Analysis presented in Figure 4 showed that there was no significant difference on improvement of knee pain (SMD = 0.05, [95%CI, -0.58 to 0.68], P = 0.88), knee stiffness (SMD= - 0.17, [95%CI, -0.76 to 0.42], P = 0.57), and physical function (SMD = -0.16, [95%CI, -0.65 to 0.33], P = 0.53) between these experimental and sham acutherapy groups. The overall result (SMD = -0.09, [95%CI, -0.40 to 0.21], P = 0.55) indicated that no obvious differences in the treatment effects existed between acutherapy and sham intervention at the same acupoints.

#### 3.2.4. Acutherapy versus Sham Acutherapy on Nonacupoints

**Figure 5:**
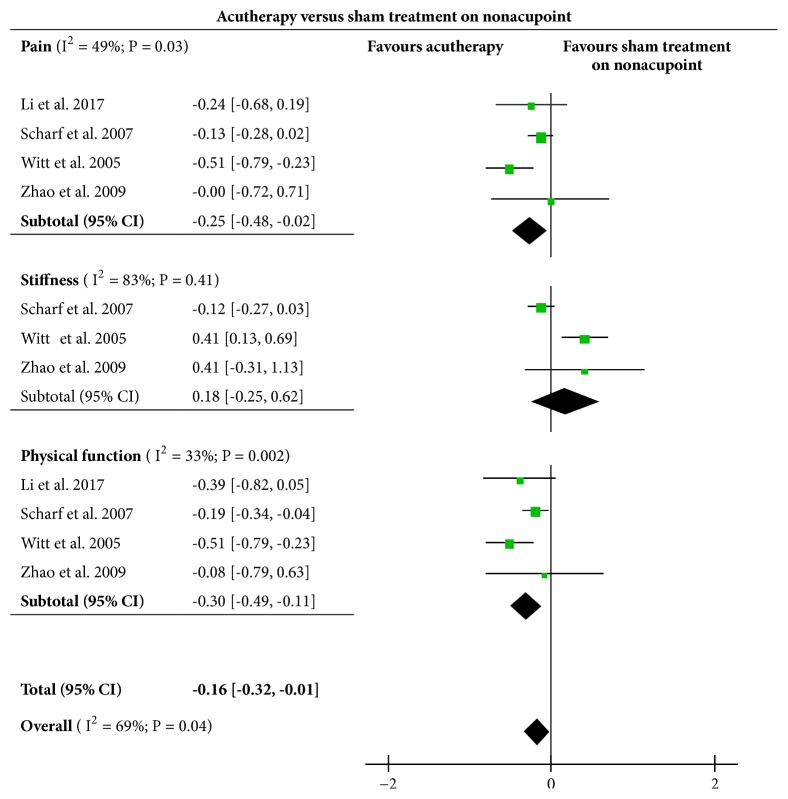
Effects of acutherapy compared with sham intervention on nonacupoints.

Four of included RCTs [10, 13, 21, 25] set up their sham groups by applying intervention on nonacupoints (points away from acupoints). The therapeutic effects were compared with experimental acutherapy groups as shown in Figure 5. Results suggested a significant difference in knee pain (SMD= -0.25, [95%CI, -0.48 to -0.02], P = 0.03) and physical function (SMD= -0.30, [95%CI, -0.49 to -0.11], P = 0.002) but not in knee stiffness (SMD=0.18, [95%CI, -0.25 to 0.62], P = 0.41). The overall results showed a significant difference in treatment effect between acutherapy and sham acutherapy on nonacupoints (SMD= -0.16, [95% CI, -0.32 to -0.01], P = 0.04) in favor of acutherapy.

### 3.3. Publication Bias and Heterogeneity

**Figure 6:**
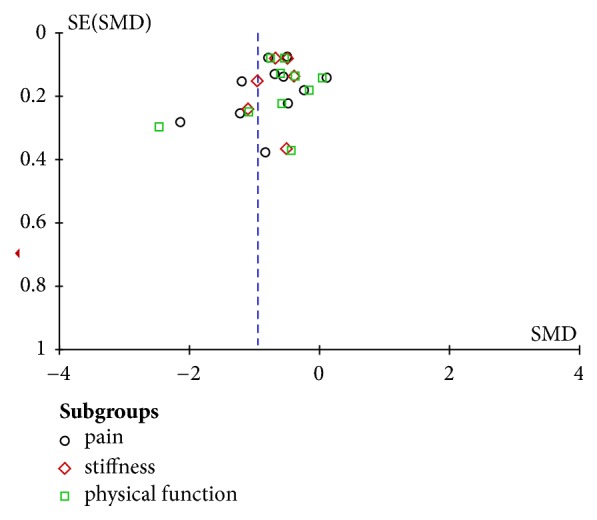
Funnel plots of the publication bias.

Publication bias evaluation was analyzed through Review Manager 5.3 involving 11 trials and 2540 subjects. As presented in the funnel plots in Figure 6, shape of the funnel plots revealed a slight asymmetric distribution, indicating a possible publication bias. Additionally, many comparisons in the meta-analysis results presented substantial (I^2^ >50%) heterogeneity in both overall and the subgroup performances.

## 4. Discussion

Current evidence extracted from the large scale and high quality RCTs were analyzed through meta-analysis. The results demonstrated that acutherapy could be an effective treatment approach for relief of symptomatic KOA in the elderly. However, its effectiveness made differences among the acutherapy groups, control groups, and sham acutherapy groups. The acutherapy presented clinical significance when compared with the usual care, but the differences were not significant as compared with the sham condition.

Compared with usual care, acutherapy showed a significant improvement in knee pain, knee stiffness, and physical function. However, no obvious improvement was observed in these parameters in sham groups, implying that both true and sham acutherapy exerted competitive effects on KOA relief. Psychological factors such as the preference and exceptions of participants could be potential reasons for these findings. The trials [10, 12, 15, 18–21, 24–26] involving both true and sham acutherapy groups did not inform subjects whether they were in an experimental or a sham group. From the perspective of the patients, they were receiving treatments on their knees and expected to have positive treatment effects. In this case, any sham intervention could produce a placebo effect. Moreover, in some three-arm RCTs [12, 15, 19, 21], more patients withdrew participation in control groups than either experimental or sham acutherapy groups and subjects even dropout immediately after group assignment. This phenomenon reflected that at least some of the KOA patients had prerandomization preferences for acutherapy and they believed that it would work no matter true or sham since they could not tell the difference.

Another explanation is that sham acutherapy does have therapeutic effects. Actually, sham group setting among those included trials could be divided into two types. The first type of sham control was to conduct intervention on the same acupoints as true acutherapy did but in a placebo way. Comparing the treatment effects of these two interventions, the data extracted from those trials [12, 15, 18–20, 24, 26] for meta-analysis indicated that there was no significant difference on treatment effect of KOA relief between true and sham acutherapy on the same acupoints. The second type of sham control was to conduct treatment on nonacupoints which have distance away from those selected acupoints used in true acutherapy [10, 13, 21, 25]. Results analyzed from these RCTs found that true acutherapy was more significantly effective for KOA relief than the sham acutherapy applied on nonacupoints. Comparison results indicated that sham interventions especially those applied on the same acupoints are as efficacious as true intervention. Thereby, this type of sham interventions such as Streitberger needle and nonmoxibustion cone on the acupoints is not real “placebo” or “sham” interventions [30, 31]. In contrast, these interventions could be regarded as new approaches or modalities derived from the conventional acupuncture treatment to deliver effective stimulation on acupoints. Therefore, acupoints could be a crucial factor for acutherapy regardless modalities through which to achieve stimulations. The most frequently used acupoints for symptomatic KOA relief summarized from included studies were the Ex-LE5 (XiYan), ST35 (DuBi), SP9 (YingLingQuan), SP 10 (XueHai), GB34 (YangLingQuan), and ST36 (ZuSanLi).

In addition, the acutherapy did not show any obvious therapeutic benefit than other physiotherapeutic approaches in KOA treatment. Three of these included RCTs [11, 15, 16] have adopted exercise-based physiotherapies (e.g., exercise oriented leg strengthening, stretching, and balance) and made comparisons with the acutherapy on the effects of KOA management. The results indicated that there was no significant difference on treatment effect in knee pain (SMD = -0.29, [95%CI, -1.20 to 0.63], P = 0.53), knee stiffness (SMD= -0.51, [95%CI, -1.23 to 0.21], P = 0.16), and physical function (SMD = -0.04 [95%CI, -0.25, 0.17], P = 0.72) between these two treatments, suggesting that both acutherapy and the adopted physiotherapeutic approaches exerted favorable effects on relief of KOA in the elderly. Thereby, for KOA management in the future, exercise-based physiotherapies could be adopted as a cost-effective adjunct to acutherapy. Moreover, other types of physiotherapeutic approach could be applied together or alternatively with acutherapy for KOA treatment that could highly improve the clinical effects.

In addition, those developed acutherapy containing more than one acuapproaches have obvious effect on treatment of KOA when compared to the corresponding sham acutherapy. This finding was contradictory to the result aforementioned that no significant effect difference existed between true and sham acutherapy. Specifically, Berman et al. [12] used acupuncture plus acuelectrical stimulation as a therapeutic approach in the experimental group while Zhao et al. [25] adopted laser acupuncture-moxibustion treatment as the experimental intervention. Compared with their sham groups, the developed acutherapy showed a significant difference in overall therapeutic effects (SMD= -0.20 [95% CI, -0.38 to -0.03], P = 0.02) over the sham acutherapy, implying that the developed acutherapy which contained more than one acuapproaches could be more effective in KOA relief than those mono-acutherapy modalities. To explore the differences on treatment effects among various acutherapy modalities, more RCTs referring to either combined or mono acutherapies should be conducted in the future. Moreover, more modalities of acutherapy could be developed to provide more alternative and complementary approaches for the elderly with KOA under various health conditions.

Although this meta-analysis presented a clear clinical relevance between different acutherapies, there remain some limitations. Firstly, high heterogeneity existed among different studies. The I square value (I^2^) above 50% indicates a high heterogeneity occurring in some overall and subgroup comparisons in this meta-analysis. The heterogeneity in comparisons may due to various reasons including different acustimulation modalities, research measures, various numbers and position selection of acupoints, different treatment periods and treatment sessions, etc. Secondly, several potential factors may lead to research bias even though methodological quality assessment was performed via Jada Scale [32] during the trial selection process. Some trials included in the present analysis were two-arm RCTs that have not built a sham group or a control group. Such group setting may bring bias to the analysis because psychological effects of acutherapy could not be excluded, and whether a sham group worked or not lacked clear elucidation. Thirdly, only end point outcomes were used for comparisons due to insufficient data. The effects of acutherapy may fluctuate at different time intervals but have not been explored in this study. At last, relevant published RCTs on acutherapy for KOA relief in the elderly are still limited. More RCTs studying on effects of various acutherapy modalities for KOA management need to be performed in the future.

## 5. Conclusion

The present study demonstrated that acutherapy could be an effective treatment approach for relieving symptomatic KOA in the elderly in terms of knee pain, knee stiffness, and physical function improvement. The intervention of sham acutherapy presented competitive effectiveness with that of true acutherapy when tested at the same acupoints; and meanwhile the intervention of sham acutherapy exerted more significant treatment effects on the tested acupoints than that on the nonacupoints. The selection of acupoints could be a crucial factor for the treatment efficacy of acutherapy. In future research, more types of acutherapy could be developed and evaluated to further determine their effects on symptomatic KOA relief in the elderly. Outcomes at more time intervals need to be recorded to explore the most suitable treatment periods and intervention sessions, and follow-up settings are warranted to be adopted by trials to examine long-term effects of various acutherapeutic methods in KOA management.

## Figures and Tables

**Table 1:** Jada scale assessment outcomes of RCTs.

Author (year)	Described as randomized?	Appropriate randomization method?	Intervention blinded to the subject?	Intervention blinded to the evaluator?	Description of withdrawals and dropouts	Score
Li et al. (2017) [10]	1/1	1/1	1/1	1/1	0/0	4/4
Write et al. (2016) [11]	1/1	1/1	0/0	0/0	1/1	3/3
Berman et al. (2004) [12]	1/1	1/1	1/1	0/0	1/1	4/4
Witt et al. (2005) [13]	1/1	1/1	1/1	0/0	1/0	4/3
Witt et al. (2006) [14]	1/1	1/1	0/0	0/0	1/1	3/3
Foster et al. (2007) [15]	1/1	1/1	1/1	1/1	1/1	5/5
Williamson et al. (2007) [16]	1/1	1/1	1/1	0/0	1/1	4/4
Berman et al. (1999) [17]	1/1	1/1	1/1	1/0	1/1	5/4
Chen et al. (2013) [18]	1/1	1/1	1/1	1/1	1/1	5/5
Mavrommatis et al. (2012) [19]	1/1	1/1	1/1	0/0	1/1	4/4
Jubb et al. (2008) [20]	1/1	1/1	1/1	0/0	1/1	4/4
Scharf et al. (2007) [21]	1/1	1/1	1/0	0/0	1/1	4/3
Manheimer et al. (2006) [22]	1/1	1/1	1/1	0/0	1/1	4/4
Kim et al. (2014) [23]	1/1	1/1	0/0	0/0	1/1	3/3
Ren et al. (2011) [24]	1/1	1/1	1/1	1/1	1/1	5/5
Zhao et al. (2009) [25]	1/1	1/1	1/1	0/0	1/1	4/4
Yurtkuran et al. (2007) [26]	1/1	1/1	1/1	1/1	1/1	5/5

Data presented as score given by author 1/score given by author 2.

**Table 2:** Characteristics of the included RCTs.

Source	Sample size	Period	Group setting (n)	Age Mean (SD)	Intervention	Main acupoints	Outcomes
Li et al. (2017) [10]	150	8 weeks	(i) acupressure (50)	71.7 (5.7)	Pressure	EX-HN3 Anmian, HT7, SP6, LIV3	Primary outcome (WOMAC ^[a]^-pain) Secondary outcome (NRS ^[b]^; WOMAC-physical function)
			(ii) sham acupressure (50)	73.2 (7.4)	Pressure	Non-acupoint	
			(iii) control (50)	73.3 (6.2)	Previous usual care	/	
Write et al. (2016) [11]	60	12 weeks	(i) group acupuncture (20)	64.7 (7.7)	Needle insertion Needle insertion +electrical stimulation	Up to 8 common points	Primary outcome (WOMAC) Secondary outcome (EQ-5D ^[c]^, OKS ^[d]^, NSAIDs ^[e]^ taking)
			(ii) individual acupuncture (20)	65.1 (9.9)			
			(iii) control (20)	64.9 (10.8)	Education	/	
Berman et al. (2004) [12]	570	26 weeks	(i) acupuncture (190)	65.2(8.4)	Needle insertion + electrical stimulation	GB34, SP9, ST36, ST35, EX-LE5, UB60, GB39, SP6, KID3	Primary outcome (WOMAC) Secondary outcome (SF-36 ^[f]^; patient global assessment; 6-minute walk time)
			(ii) sham acupuncture (191)	66.2 (8.7)	Nonpenetrating needle	Same acupoints	
			(iii) control (189)	65.1 (8.8)	Arthritis education	/	
Witt et al. (2005) [13]	300	8 weeks	(i) acupuncture (150)	64·5 (6·4)	Needle insertion	ST34, ST35, ST36; SP9, SP10; BL40; KID 10; GB33, GB34; LIV8	Primary outcome (WOMAC) Secondary outcome (VAS ^[g]^; PDI ^[h]^; SF-36; SES ^[i]^; HAD ^[j]^)
			(ii) sham acupuncture (76)	63.4 (6.6)	Needle insertion	non-acupoints	
			(iii) control (74)	63.6 (6.7)	Previous usual care	/	
Witt et al. (2006) [14]	712	3 months	(i) acupuncture (357)	60.6 (10.2)	Needle insertion	Selected by physician	Primary outcome (WOMAC) Secondary outcome (SF-36)
			(ii) control (355)	61.9 (10.6)	Previous usual care	/	
Foster et al. (2007) [15]	352	12 months	(i) acupuncture +advice & exercise (117)	63.1 (8.7)	Needle insertion + advice and exercise	SP9, SP10, ST34, ST35, ST36, EX-LE5, GB34, trigger points.	Primary outcome (WOMAC-pain) Secondary outcome (WOMAC; participant global assessment)
			(ii) sham acupuncture +advice & exercise (119)	62.8 (9.4)	Non-penetrating needle + advice & exercise	Same acupoints	
			(iii) control (116)	63.8 (8.3)	Previous usual care	/	
Williamson et al. (2007) [16]	181	6 weeks	(i) acupuncture (60)	72.4 (7.71)	Needles insertion	SP10, EX-LE5, ST35, ST36, SP9, GB34, LIV3	Primary outcome (OKS) Secondary outcome (WOMAC; VAS; HAD);
			(ii) physiotherapy (60)	70.0 (8.79)	Exercises	/	
			(iii) control (61)	69.6 (10)	Advice	/	
Berman et al. (1999) [17]	73	12 weeks	(i) acupuncture (37)	65.7 (7.95)	Needle insertion + electrical stimulation	GB34, SP9, ST36, ST35, EX-LE5, UB60, GB39, SP6, KID3	WOMAC Lequesne indices
			(ii) control (36)	65.5 (9.13)	Previous usual care	/	
Chen et al. (2013) [18]	213	12 weeks	(i) acupuncture (104)	60.5 (11.1)	Needle insertion	GB34, SP9, ST 36, ST 35, EX-LE5	Primary outcome (WOMAC) Secondary outcome (BPI ^[k]^; SF-36; Patient Global Impression of ChangeL; 6-min walk distance
			(ii) sham acupuncture (109)	60.4 (11.7)	Non-penetrating needle	Same acupoints	
Mavrommatis et al. (2012) [19]	120	8 weeks	(i) acupuncture (40)	62.3 (9.9)	Needle insertion + etoricoxib	ST36, SP9, SP10, GB34, EX-LE 2, Ex-LE5	Primary outcome (WOMAC) Secondary outcome (SF-36; VAS; Algometer)
			(ii) sham acupuncture (40)	60.1(11.1)	Non-penetrating needle + etoricoxib	Same acupoints	
			(iii) control (40)	63 (10.6)	Etoricoxib only	/	
Jubb et al. (2008) [20]	68	5 weeks	(i) acupuncture (34)	64.1 (1.6)	Needle insertion	LI4, SP10, Ex-LE5, SP9, GB34, ST 36, LIV 3, BL 40, BL 57.	Primary outcome (WOMAC-pain) Secondary outcome (WOMAC; VAS)
			(ii) sham acupuncture (34)	66.1 (1.9)	Non-penetrating needle	Same acupoints	
Scharf et al. (2007) [21]	1039	26 weeks	(i) acupuncture (330)	62.8 (9.9)	Needle insertion	ST34, ST36, EX-LE5, SP9, SP10, GB34	Primary outcome (WOMAC) Secondary outcome (SF-12 ^[l]^; global patient assessment)
			(ii) sham acupuncture (367)	63.0 (10.1)	Non-penetrating needle	Non-acupoints	
			(iii) control (342)	62.6 (10.1)	Previous usual care	/	
Manheimer et al. (2006) [22]	570	26 weeks	(i) acupuncture (190)	65.2 (8.4)	Needle insertion	GB34, SP9, ST36, ST35 BL60, GB39, SP6, KI3	Primary outcome (WOMAC-pain & function) Secondary outcome (patient global assessment; 6-minute walk distance; SF-36)
			(ii) sham acupuncture (190)	66.2 (8.7)	Non-penetrating needle	Same acupoints	
			(iii) control (190)	65.1 (8.8)	Education	/	
Kim et al. (2014) [23]	212	13 weeks	(i) moxibustion (102)	56 (/)	Burn moxibustion cone	ST36, ST35, ST34, SP9, Ex-LE5, SP10	Primary outcome (WOMAC; SF-36; BDI ^[m]^; physical performance)
			(ii) control (110)	57 (/)	Previous usual care	/	Secondary outcome (C-reactive protein; Erythrocyte sedimentation rate
Ren et al. (2011) [24]	59	6 weeks	(i) moxibustion (31)	64.03 (7.2)	Burn moxibustion cone	EX-LE 5, ST35, trigger points Same acupoints	WOMAC; temperature
			(ii) sham moxibustion (28)	62.57 (8.12)	Burn non-moxibustion cone		
Zhao et al. (2009) [25]	40	4 weeks	(i) laser acupuncture (20)	60.10 (6.83 )	Laser stimulation on acupoint	ST 35	Primary outcome (WOMAC) Secondary outcome (patient global assessment; medication usage; masking effectiveness; adverse effect)
			(ii) sham laser acupuncture (20)	59.40 (6.15)	Laser intervention on non-acupoint	2 cm from ST35	
Yurtkuran et al. (2007) [26]	55	2 weeks	(i) laser acupuncture (27)	51.83 ± 6.83	Laser stimulation	SP9	Primary outcome (VAS; 50 foot w ^[n]^; KC ^[o]^; MTS ^[p]^; WOMAC) Secondary outcome (Quality of life)
			(ii) sham laser acupuncture (26)	53.48 (7.13)	Red light stimulation	Same acupoints	

[a] WOMAC: Western Ontario and McMaster Universities Osteoarthritis Index; [b] NRS: the numeric rating scale; [c] EQ-5D: EuroQol five-dimension quality of life instrument; [d] OKS: Oxford Knee Score; [e] NSAIDs: nonsteroidal anti-inflammatory drugs; [f] SF-36: The Short Form (36) Health Survey; [g] VAS: visual analogue scale; [h] PDI: pain disability index; [i] SES: questionnaire for assessing the emotional aspects of pain; [j] ADS: Hospital Anxiety and Depression Scale; [k] BPI: Brief Pain Inventory; [l] SF-12: 12-Item Short Form Health Survey; [m] BDI: Beck Depression Inventory; [n] 50 foot w: 50-foot walking time; [o] KC: knee circumference; [p]MTS: Medial Tenderness Score.
